# Textile Supplies for Hospitals

**Published:** 1916-02-05

**Authors:** 


### TEXTILE SUPPLIES FOR HOSPITALS

### IV. The Animal Fibres.

The error most frequently made in regard to wool is that of considering it hastily as all alike. It does no harm, at least, to recognise that wools are extraordinarily various. The fleece of one and the same sheep is distinctly different in its several parts. Every breed of sheep produces wool somewhat different from every other, and wool is so modified by the soil, keep, and climate that every sheep-raising district, and still more every sheep-growing country, leaves its reflection upon the wool produced in it. The differences range from the slight to the profound. There are in this country breeds producing wool four times the length of certain other breeds, and it is a simple truism that in diameter the difference between wools not respectively the finest and coarsest that would be chosen is as ten to one. The facts may at any rate serve as a warning against regarding all wool as the same thing. Wools are alike, however, in coming from the sheep or from one of its near relations, and in sharing a certain relationship to hair, feathers, and horn. They differ from hair not necessarily in being finer, but in being less smooth and straight. Cotton fibres derive their binding power from the presence of their natural twist, and linen fibres from a roughness of surface, while fibres of wool owe their cohesion largely to the minute scales projecting from their edges and rendered visible by the microscope. Wool ceases to be such and gravitates into hair as these imbrications decrease in frequency, and it becomes at the same time a less and less manageable fibre for purposes of manufacture. As well as these serrations wool shows a more manifest crimpiness or waviness, and the finer the wool in diameter the more of those natural curls occur in the inch. Both are to be seen with more advantage in the raw sample than in fibre detached from finished goods, but in some measure the twin features remain.

Chemically, wool is a complex of carbon, hydrogen, nitrogen, oxygen, and sulphur, with the 2 to 3 per cent. of sulphur occupying a central place, apparently, in the structure. At all events wool is quickly disintegrated by reagents, like the caustic alkalies, that combine with the sulphur. Wool is dissolved in a few minutes in a caustic-soda solution of 5 per cent., and it is injured in one degree or another by every application of strongly alkaline soap. The affinity of wool with sulphur, upon the other hand, allows woollens to be stoved with general impunity in sulphur fumes, which in the presence of moisture cannot safely be applied to cotton or linen goods. Oxydising down to sulphuric acid, the solution of sulphur dioxide chars vegetable fibre, and, indeed, sulphuric acid is the chemical commonly employed in manufacture to carbonise and destroy vestiges of vegetable fibres remaining in wool or in woollen rags.

### Sheep's Wool.

The essential differences between wools of distinct classes single them out for special purposes, so that the wool adapted to maintain an erect pile in carpets, for example, would not be selected to wear next the skin, or for fabrics requiring the use of fine-spun yarn. A fairly wide variety of wools are used in making flannels of all qualities, but the preference is for short, curly wools of good felting power. The short wools are used also for blankets, which are made very largely from the shorter fibres combed out of the wools used in the worsted process of manufacturing. The longer of the fibres from which these so-called "noils" are taken for such purposes as blanket-making may serve, for instance, for a nurse's worsted cape. Some wools, classified as "combing" quality, are suited for worsted preparation, in course of which the short stuff is removed and the long fibres are laid parallel; others catalogued as "clothing," only for preparation by carding, in which process long and short are intermixed and every opportunity is taken to interlock the serratures of the neighbouring fibres one with another. The suitability of the specimens for one purpose or the other is a matter as much of the capacity of machines as of anything, and no hard-and-fast distinctions can be laid down.

The chief kinds of wool admit of an easy classification. The finest is from the merino sheep, preponderantly produced in Australia, and wool of this quality goes in some conditions under the alternative names "Botany" and "Saxony." The Americans call it expressively "blood" wool, in the same way as thoroughbred horseflesh is styled bloodstock. Medium wools, known as "crossbred," are more plentiful and cheaper than merino, and are produced upon animals with an ancestry of crossed merino blood. New Zealand and Argentina are the most prolific sources of crossbred. These wools are of a length and fineness varying with their parentage, and the description is an exceedingly broad one. The other parent of the crossbred sheep is normally one of the English breeds, be it a big-bodied, long-woolled Lincoln or Leicester, or a short-woolled animal like the Southdown. Additional to these are the coarse or low wools of many varieties, and capable in general only of humbler uses. The merino sheep is not uneatable, but it is a breed grown for its wool more than for its mutton, and the crossbred and English varieties are, on the contrary, bred rather for their mutton than their wools. Textile terminology abounds in inconsistencies, and it may be worth while to remark that "merino" applied to textile goods does not under all circumstances connote manufacture from wool of merino breed. In the accepted usage of the hosiery business the term stands for a mixture of wool and cotton from which quantities of undergarments are made.

Note.—Previous articles in this series appeared on December 18, 1915, and January 8 and 22, 1916. The next article will deal with "Manufacturing Methods and Processes."

### Goat and Camel Hair.

A kindred distortion may be noticed in the word "cashmere," variously applied to light dress-stuffs and to fashionable trouserings made with merino wool and owning no actual connection with the Cashmere goat, famous as a provider of material for costly shawls. Hair of this goat is used chiefly for making warm and beautifully soft knitted garments, belonging rather to the order of private comforts than of hospital supplies, and this goat is one of several animals providing hair like enough to wool to serve a kindred purpose. Of these last the Angora goat, the producer of mohair, is of the most importance, and its fine, glossy fleece furnishes at a quite moderate cost materials for dresses and light cloaks that have more than good wearing properties to recommend them. Mohair dress-stuffs, woven with a cotton warp, have a natural stiffness which forbids them to drape as elegantly as stuffs made from short, fine wool, but makes them much less liable to harbour dust. In the popular mind mohair is confused inextricably with alpaca, a hair grown on the Peruvian llama, an animal intermediate between a goat and a camel. Alpaca is somewhat finer and more slippery than mohair, but it is parti-coloured and barely procurable in cloth in any other colour than black, whereas mohair is naturally white or yellowish, and may be dyed to all colours. Camel hair is used for the same purposes as cashmere, and notably for articles of the Jaeger clothing type. The soft undergrowth of the camel and the Cashmere goat and not the coarse beard-hairs are taken for fine textiles. Camel-hair is of a natural fawn and cashmere of a prevalently bluish cast, and both are employed generally in the undyed state. It cannot, however, be deduced that all fawn-coloured articles or blue-grey ones are respectively camel or cashmere fleece. The colourings are imitated by resort to dyeing, just as "natural" wool garments are mostly dyed ones. In the beginning "natural" perhaps implied the intermixture of wool from black or brown sheep with wool from white, and this blend was conceived to be superior in that dye-stuff was not brought next the skin and that the colours did not change. The demand for "natural" underwear far outdistances the best efforts of coloured sheep.

### Horse-hair and Other Stuffings.

The cheapest coloured blankets, some low-class knitted articles, and cheap portières prove to contain calf-hair recovered from the tan-yards, but cow-hair is a minor fibre at most. A more immediately interesting raw article is horse-hair in its capacity not as a component of cloth, but as a stuffing. The tail hair of horses palpably is capable of being woven into hard-wearing seating material, and the business of weaving isolated horse-hairs across a cotton warp in a hand-loom is one of the surviving handicrafts of the textile industry. Horse-hair is woven, too, into the stiffening interlinings used by tailors. For mattresses the tail hair is used and is curled by twisting it into a rope, soaking it in water and baking it for twelve hours at 350° F., so that the hair thus acquires a permanent set.

In the common experience of hospitals, horse-hair stuffing, although so much the most expensive in the first instance, is the most economical in view of wear and tear and repeated disinfections. Wool flocks are not quite the only possible alternative to hair. Mattresses may be, and are, stuffed in some countries with sheep's wool, and coarse wools like our Highland blackfaced have been used for the purpose, receiving first a preliminary scouring and teasing. The fact is mentioned not as a recommendation of an alternative material, but in course of a passing reference to the nature of wool flocks. Only in a modified sense are flocks raw material. They are a by-product of the manufacture of cloth, and represent very short fibre incidentally removed in washing, cutting, brushing or otherwise treating woollen cloth or blankets; or, alternatively, they are rag flocks, the direct outcome of the disintegration of woollen rags into fibre again. They have an origin either in new or in old cloth, and the old cloths made into flocks are the least respectable of their kind, exhibiting a fibre too poor for re-manufacture, and normally very dirty.

### Uses of Silk.

A few English hospitals make some use of silk fibre as a stuffing, although in wear the fibres are apt to mat and form lumps. The silk noils employed are at all events cheap and clean, and as their provenance is somewhat distant, it may be briefly traced. The cocoon whence all silk comes is softened in warm water and unwound. Ends of the fibre from several cocoons unwound simultaneously are run loosely together, and the combined thread thus formed is the raw silk of commerce. By further winding, twisting, selection, and rejection, the raw becomes thrown silk and organzine for surgical and other uses. One half or so of the total weight of the cocoons being ineligible for unwinding in the basins of the silk *filature* is bulked together and sold at a fairly high price under the name of silk waste. This waste is bought by silk-spinners, who treat it in broadly the same manner as linen, worsted, or cotton. It is boiled to remove the animal gum with which it is coagulated, and is put through a series of dressing operations designed to remove the longer and hence more valuable fibres. A residue remains, and this clean, soft silk sells at a few pence per pound, mainly to those who have machinery for turning it into a thick silk yarn. These noils are the waste of silk waste, not necessarily free from small vegetable impurities or dried portions of silkworm, but beautiful in lustre and softness. The material has little affinity for water although it has a marked capacity for absorbing oil, and upon this account the inferior silk noils are used largely to make wiping cloths for machinery. Oily cotton cloths lying about present a danger of spontaneous combustion which is appreciably reduced when non-flammable silk wiping cloths are used—as they commonly are in textile mills.

